# The RIPper, a web-based tool for genome-wide quantification of Repeat-Induced Point (RIP) mutations

**DOI:** 10.7717/peerj.7447

**Published:** 2019-08-26

**Authors:** Stephanie van Wyk, Christopher H. Harrison, Brenda D. Wingfield, Lieschen De Vos, Nicolaas A. van der Merwe, Emma T. Steenkamp

**Affiliations:** 1 Department of Biochemistry, Genetics and Microbiology, Forestry and Agricultural Biotechnology Institute (FABI), University of Pretoria, Pretoria, Gauteng, South Africa; 2 Department of product and software development, Amplo PTY, Pretoria, Gauteng, South Africa

**Keywords:** Repeat-Induced Point mutations, RIP, RIP profile, Large RIP affected regions, Fine-scale RIP analyses, Genome-wide quantification, Web-based tool, The RIPper

## Abstract

**Background:**

The RIPper (http://theripper.hawk.rocks) is a set of web-based tools designed for analyses of Repeat-Induced Point (RIP) mutations in the genome sequences of Ascomycota. The RIP pathway is a fungal genome defense mechanism that is aimed at identifying repeated and duplicated motifs, into which it then introduces cytosine to thymine transition mutations. RIP thus serves to deactivate and counteract the deleterious consequences of selfish or mobile DNA elements in fungal genomes. The occurrence, genetic context and frequency of RIP mutations are widely used to assess the activity of this pathway in genomic regions of interest. Here, we present a bioinformatics tool that is specifically fashioned to automate the investigation of changes in RIP product and substrate nucleotide frequencies in fungal genomes.

**Results:**

We demonstrated the ability of The RIPper to detect the occurrence and extent of RIP mutations in known RIP affected sequences. Specifically, a sliding window approach was used to perform genome-wide RIP analysis on the genome assembly of *Neurospora crassa*. Additionally, fine-scale analysis with The RIPper showed that gene regions and transposable element sequences, previously determined to be affected by RIP, were indeed characterized by high frequencies of RIP mutations. Data generated using this software further showed that large proportions of the *N. crassa* genome constitutes RIP mutations with extensively affected regions displaying reduced GC content. The RIPper was further useful for investigating and visualizing changes in RIP mutations across the length of sequences of interest, allowing for fine-scale analyses.

**Conclusion:**

This software identified RIP targeted genomic regions and provided RIP statistics for an entire genome assembly, including the genomic proportion affected by RIP. Here, we present The RIPper as an efficient tool for genome-wide RIP analyses.

## Introduction

The Repeat-Induced Point (RIP) mutation pathway is a genome defense mechanism that has evolved exclusively in fungi (Gladyshev, 2017). This pathway is thought to operate only during sexual reproduction and serves to preserve and protect fungal genome integrity from mobile genetic elements such as transposable elements (TEs) that typically contain repeated sequences (Selker et al., 1987; Selker, 1990; Hane et al., 2015). RIP distinguishes genetic targets based on shared homology between the repeats and permanently mutates them by inducing cytosine to thymine transition mutations (Selker et al., 1987). This mutational process is coupled with epigenetic silencing of the RIP affected regions (via methylation of the remaining cytosine residues) to further attenuate the deleterious effects of TEs (Selker et al., 2003). Unlike other eukaryotic defense mechanisms, RIP has no other known biological functions and is solely directed to counteract the actions of TEs and other duplicated regions (Hane et al., 2015).

RIP introduces cytosine to thymine transition mutations in duplicated and repeated sequences (Selker et al., 1987). Transition mutations caused by RIP are typically considered in relation to their dinucleotide context (Selker et al., 2003; Lewis et al., 2009; Margolin et al., 1998). For instance, pre-RIP affected sequences, or sequences that are not targeted by RIP, are typically more GC-rich (e.g., the dinucleotides CpA, TpG, ApC, GpT) whereas RIP affected sequences are rich in AT dinucleotide sequences (ApT, TpA). The genomic regions altered by RIP therefore display an increase in AT composition (Selker et al., 2003). Accordingly, *in silico* assessment of RIP is based on measuring changes in the frequency of RIP targeted dinucleotides in relation to the resulting RIP product dinucleotides (Selker et al., 2003; Lewis et al., 2009; Margolin et al., 1998; Hane & Oliver, 2008). A depletion of appropriate RIP dinucleotide substrates, coupled with an increase in RIP dinucleotide products, in close proximity to one another, is the hallmark of RIP.

*In silico* investigation of RIP have greatly aided the current understanding of the nature of RIP mutations in genes, TEs and other repeated sequences (Hane & Oliver, 2008). For example, alignment-based RIP analyses of repeated motifs enabled the identification of a dominant RIP mutation form for a particular class of a repeated sequences (Hane & Oliver, 2008), and facilitated ancestral reconstruction of RIP affected regions to their original, pre-RIP forms (Hane & Oliver, 2010). These analyses of specific duplicated sequences and TEs have also improved our understanding of the RIP pathway’s taxonomic range within the phylum Ascomycota (Clutterbuck, 2011). However, few previous studies have explored the effects of RIP on a genome-wide scale. Some have measured changes in the standard RIP indices for genomic regions of interest using, for example, RIPCAL (Selker, 1990; Selker et al., 2003; Lewis et al., 2009; Margolin et al., 1998; Li et al., 2017; Rouxel et al., 2011). However, many studies depend on estimations of RIP inferred using indirect or surrogate methods that measure the consequences of RIP (e.g., GC content depletion) across a genome using software such as OcculterCut (Testa, Oliver & Hane, 2016; Li et al., 2017; Rouxel et al., 2011).

Although the impact of RIP’s activity on genome evolution remains poorly understood, RIP mutations are known to “leak” into neighboring regions where they affect single copy regions (Rouxel et al., 2011). Various authors have suggested that such mutation of unintended genetic targets may have served as a source of genetic variation and have contributed to the evolution of various aspects of the fungal lifestyle, including host range and pathogenicity (Fudal et al., 2009; Meerupati et al., 2013). However, this “off-target” effect of RIP has not been investigated in many fungi, because RIP is usually studied within the bounds of duplicated and/or TE genomic sequences. Moreover, RIP allows the formation of long stretches of AT-rich regions that have a reduced coding capacity, are lineage-specific, and may constitute large proportions of fungal genomes (Fudal et al., 2009; Hamann, Feller & Osiewacz, 2000). In this way, RIP likely also plays an important role in the evolution of genome architecture of many fungi. There is thus a need to investigate RIP on a genome-wide scale in order to identify regions that are extensively affected by RIP and to understand in what manner RIP contributes to both genome evolution and pathogen development. Automation of genome-wide RIP mutation analysis will undoubtedly aid such investigations.

Here, we present a web-based tool, The RIPper, which allows for genome-wide quantification and identification of RIP mutations and also generates detailed RIP profiles. Data generated by The RIPper further allows for fine-scale RIP analyses of genomic regions of interest and visualization of changes in RIP index values relative to GC content changes, while it also provides summary statistics on RIP. This tool further identifies Large RIP Affected Regions (LRARs) across genome sequences by using an innovative approach to detect genomic regions significantly impacted by RIP. We demonstrate The RIPper’s ability to detect RIP in fungal sequences by performing RIP analyses on the genome assembly of *Neurospora crassa*, as well as on gene regions, TEs, and large genomic regions previously shown to be affected by RIP.

## Methods

### The RIPper

The RIPper includes an open source set of tools that allows for genome-wide quantification of RIP mutations in the genomes of Ascomycota. The RIPper accepts whole genome sequence data in FASTA format. This program can be used as an online tool (http://theripper.hawk.rocks) or it can function as a stand-alone executable file using Windows, Mac or Linux operating systems. The RIPper is also available on github (https://github.com/TheRIPper-Fungi/TheRIPper).

The RIPper is a web-based bioinformatics tool that was built using Asp.Net Core 2.1. It uses Entity Framework as its database together with Object Relational Mapping (ORM). Furthermore, the .Net bio library is used for loading and handling FASTA files. The server used for the online version of The RIPper uses a PostgreSQL database to handle multiple concurrent users. The standalone version uses SQLite. Additionally, The RIPper is compatible with all commonly used web browsers including Google Chrome, Mozilla Firefox and Microsoft Edge.

The design of The RIPper is based on the principles of measuring changes in RIP product and substrate dinucleotide frequencies using RIP indices (Selker et al., 2003; Lewis et al., 2009; Margolin et al., 1998). The frequency of dinucleotides that are recognized as suitable genetic targets of the RIP pathway are calculated using the RIP substrate index (Selker, 1990; Lewis et al., 2009), while the products produced are calculated using the RIP product index (Lewis et al., 2009; Margolin et al., 1998). The RIP composite index reflects changes in both RIP product and RIP substrate dinucleotide frequencies (Lewis et al., 2009). The RIPper calculates changes in the RIP index values and the average GC content using a sliding window approach (e.g., Table S1). The three indices are determined as indicated below, and windows with index values of *x* suggest that the region is affected by RIP. These RIP index values for *x* were previously published (Selker, 1990; Selker et al., 2003; Lewis et al., 2009; Margolin et al., 1998), but are additionally adjustable according to user preferences.

Product index value: $ \frac{TpA}{ApT} $: 0.8 <  *x* ≥ 1.1

Substrate index value: $ \frac{CpA+TpG}{ApC+GpT} $: 0.9  ≥ *x*

Composite index value: $[ \frac{TpA}{ApT} ]\text{-}[ \frac{CpA+TpG}{ApC+GpT} ]$: *x* > 0

The GC content of each window is calculated as follows:

GC content: $ \frac{G+C}{G+C+A+T} $

### Analyses and outputs of The RIPper

In a standard analysis, the genome-wide impact of RIP is quantified by comparing the total number of windows indicating RIP-positive index values against the total number of windows investigated for the entire genome sequence. For a window to be considered RIP-positive, all three RIP indices should indicate RIP activity. The window size for the RIP index analyses is user defined, but default parameters for genome-wide quantification is pre-set at 1,000 bp with a 500 bp step size (e.g., Table S1).

**Figure 1:**
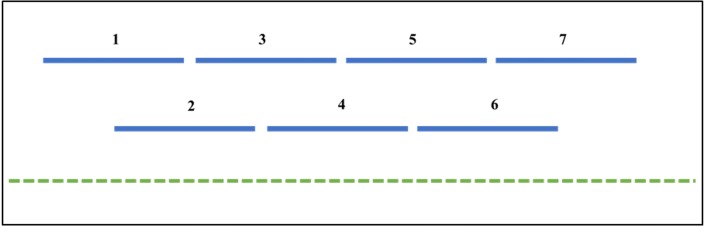
The RIPper identifies putative Large RIP Affected Regions (LRARs) in genomic sequences. The default definition of an LRAR is a region of at least 4,000 base pairs (bp) affected by RIP (as indicated by the RIP product, substrate and composite indices). Such a 4000 bp region is made up of seven consecutive 1,000 bp sliding windows (500 bp step size). The blue lines represent the sliding windows investigated across the length of a genomic sequence (green dotted line).

The RIPper can identify large genomic regions that are putatively extensively affected by RIP. The criteria for LRARs is adjustable according to user preferences, but default parameters constitute seven consecutive RIP-positive windows, spanning at least 4000 bp (Fig. 1). An index chain describes the total number of consecutive RIP-positive windows needed to define a LRAR. For more stringent analysis, the RIP index values considered for each window can be adjusted. The data generated using the ‘Calculate LRAR’ tool provides information on the genomic location, the total number of consecutive RIP-positive windows, average RIP index values across the length of the LRAR and the average GC content of each LRAR identified.

**Figure 2:**
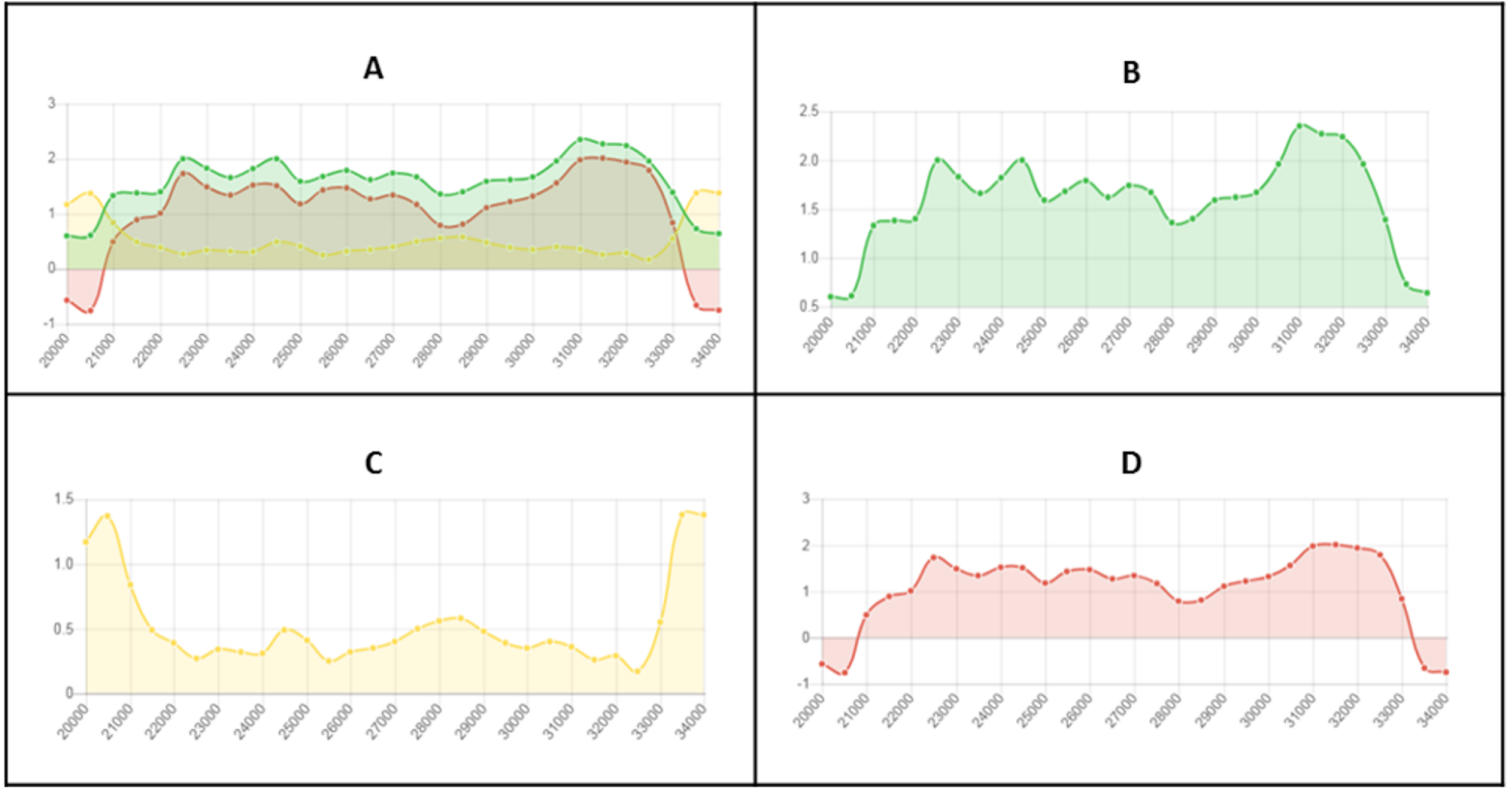
Example of fine-scale changes in RIP index values. Fine-scale changes in RIP index values for the first Large RIP Affected Region (LRAR) identified on the first chromosome/linkage group of *Neurospora crassa* strain OR74a (NCBI accession number GCF_000182925.2/). (A) Changes in RIP index values are represented on the *y*-axis and the nucleotide position on the sequence is indicated on the *x*-axis. RIP index values depicted: RIP product (green), RIP substrate (yellow) and RIP composite (red) (B) RIP product index values above 1.1 are indicative of RIP activity. (C) RIP substrate index values below 0.75 are indicative of RIP activity, and (D) RIP composite index values exceeding 0 are indicative of RIP activity. RIP analyses were performed using a 1,000 bp window size and 500 bp step size.

Another important feature of The RIPper is its ability to visualize changes in RIP index values across the length of a genomic sequence. Changes in RIP composite, product and substrate indices are provided for each window (Fig. 2). This tool further allows the user to browse through the query sequence based on a user defined range. Furthermore, changes in RIP index values, the total number of LRARs and LRAR statistics for each scaffold or chromosome within a genome assembly, can be considered individually.

The RIPper summarizes the statistics for a defined set of genomic sequences in a RIP profile. The RIP profile includes statistics on the average RIP product, substrate, and composite index values, as well as the average GC content for all LRARs identified. The RIP profile further provides information on the total size of the genome affected by RIP, its average GC content, and the total number of windows investigated.

All information generated using The RIPper is downloadable in Excel, CSV and PDF format. The parameters for determining RIP index values and GC content (calculated for each window and each step window) are all user-defined. The summary of RIP statistics is also downloadable as a RIP profile in Excel format. Fine-scale RIP analyses figures produced by The RIPper are downloadable in Portable Graphics format (PNG).

### Potential for erroneously identifying RIP affected regions

GC content is one of the major factors that might cause incorrect identification of a sequence as being affected by RIP (i.e., false positives results). To investigate this issue, simulated nucleic acid sequences were generated and subjected to RIP analyses. Each simulated sequence was one million base pairs (Mbp) in size and was generated using the Random DNA Generator (http://www.faculty.ucr.edu/ mmaduro/random.htm) software. Nine sets (each containing 100 sequences) were generated, where each set contained sequences with an average GC content of respectively 10%, 20%, 30% through to 90%. All simulated sequences were subjected to full RIP analysis with The RIPper using a 1,000 base pair (bp) sliding window with a 500 bp step size. The RIP substrate index values evaluated were 0.75, 0.8, 0.85 and 0.9, while the RIP product index values evaluated were 1.1, 1.15, 1.2 and 1.25.

These ranges of RIP index values were also evaluated using the genome sequences of organisms known to be either RIP capable or incapable. These included *Neurospora crassa* strain OR74a, *Trichoderma reesei* strain QM6a and *Leptosphaeria maculans* strain JN3, which have all been experimentally verified to be RIP capable (Selker et al., 2003; Li et al., 2017; Fudal et al., 2009). To represent genomes in which RIP is not possible, we utilized those of the ascomycete yeast *Candida albicans* strain SC5314, the microsporidian fungus *Encephalitozoon cuniculi* strain GB-M1 and the bacterium *Escherichia coli* strain K-12. Experimental studies showed that *C. albicans* is not RIP capable (Clutterbuck, 2011), while Microsporidia diverged prior to the evolution of the RIP pathway (Horns et al., 2012) and bacteria cannot acquire cytosine and thymine transition mutations via RIP. All six genome sequences were obtained from the database of the National Centre for Biotechnology Information (NCBI; https://www.ncbi.nlm.nih.gov) using the accession numbers GCA_000182965.3, GCF_000091225.1, GCF_000005845.2, GCA_000230375.1, GCA_000182925.2 and GCA_002006585.1. As before, analyses were done using the range of values for the RIP substrate and product indices.

### Validation of RIP analyses using The RIPper

The RIPper was used to determine the occurrence and frequency of RIP mutations in sequences that were experimentally and computationally shown to be affected by RIP.These included particular regions (ranging from 478 to 61,000 bp in size) in the genomes of *L. maculans* (Plissonneau et al., 2016), *N. crassa* (Margolin et al., 1998), *Podospora anserina* (Hamann, Feller & Osiewacz, 2000), *Colletotrichum cereale* (Crouch et al., 2008), *Aspergillus fumigatus* (Paris & Latgé, 2001), *Aspergillus oryzae* (Montiel, Lee & Archer, 2006), *Magnaporthe grisea* (Nakayashiki et al., 1999) and *Chrysoporthe deuterocubensis* (Kanzi et al., 2019). We also used the genomes of *N. crassa* strain OR74a and *T. reesei* strain QM6a, which are known to be RIP competent (Selker et al., 2003; Li et al., 2017). All these sequences were obtained from the NCBI database using accession numbers GCF_000182925.2/, GCA_002006585.1, KT804641.1, AF181821.1, AJ270953.1, DQ663509.1, AF202956.1, DQ327733.1, AB024423.1 and GCF_001513825.1.

The RIPper was used to determine whether this software was capable of identifying large regions affected by RIP in the sequences analyzed. Additionally, for the *N. crassa* (OR74a) genome assembly, we also generated genome-wide RIP statistics using the “RIP profile” tool. The genomic locations and RIP statistics of putative LRARs were determined using the “calculate LRAR” tool, while the proportion of individual *N. crassa* chromosomes affected by RIP were calculated using the “RIP sequence” tool. Also, for the *T. reesei* (QM6a) genome, chromosome-wide changes in RIP index values were compared to changes in GC content, as it was previously shown that centromeric regions are AT-rich due to RIP mutations (Li et al., 2017).

All chromosome- and genome-wide analyses used sliding windows of 1,000 bp and 500 bp steps. For particular genomic regions, fine-scale analyses were performed using 100 bp sliding windows with a step size of 50 bp. The genomic sequence containing the mating type region of *C. deuterocubensis* was analyzed using a 1,000 bp window and a 500 bp step size. The RIPper was used to generate graphs for visualizing changes in RIP index values and GC content across the length of the query sequences. This software was also used to generate RIP summary statistics.

## Results

### Potential for erroneously identifying RIP affected regions

To investigate the influence of GC content composition on RIP statistics, simulated sequences consisting of different GC content ranges were subjected to RIP analysis using varying degrees of stringency of the RIP parameters. The overall results showed that less stringent RIP parameters (i.e., lower RIP product index values and higher RIP substrate values) more frequently led to the identification of false positives in the simulated datasets (Table S2 and Fig. S1). RIP analyses performed under the least stringent conditions (RIP product index value of 1.1 and RIP substrate index value of 0.9), identified RIP affected regions in 656 data sets, while those done with the most stringent parameters (RIP substrate index value of 0.75) allowed detection of RIP mutations in only two of the 900 simulated sequences (Fig. S1). Both of these sequences had an average GC content of 80%, where 6% of the sequences apparently consisted of RIP affected regions. However, no LRARs were identified in any of the simulated data sets (900 simulations).

Notably, at less stringent RIP substrate index values (0.8 to 0.9), more than 1% of the simulated data sets were suggested to be affected by RIP (Table S2). The occurrence and frequency of RIP mutations were more often recorded in data sets composed of high GC contents (Table S2; 50–90%) under less stringent RIP substrate parameters. Also, the frequency of erroneous identification of RIP mutations was much higher in data sets with >70% GC. This was particularly true when less stringent RIP substrate values (0.8 to 0.9) were used.

Analysis of the simulated data sets showed that stringent RIP substrate index parameters are needed to minimize the possibility of erroneous identification of RIP mutations. Our results showed that the most optimal parameter for limiting or excluding the occurrence of false positives is to use a RIP substrate index value of 0.75 (Fig. S1). Changes in RIP product value cut-offs generally had little effect on the RIP statistics, but fewer false positives were obtained using a RIP product cut-off value of 1.1.

We also investigated whether the stringency of RIP index parameters could potentially influence the outcome of analyses performed on the genome sequences of organisms known to be either RIP capable or incapable (Table S3). By making use of these two sets of genomes, it was thus possible to contrast the effects of parameter stringency in genomes where RIP is possible (positive controls; i.e., *N. crassa, L. maculans* and *T. reesei*) and genomes where RIP is impossible (negative controls; i.e., *E. coli*, *C. albicans* and *E. cuniculi*). Accordingly, under the most stringent RIP substrate parameter applied (0.75), no RIP positive windows were recorded in any of the negative controls (except for a single window in the genome of *E*. *cuniculi*). At this stringency level, numerous RIP positive windows and LRARs were detected in the genomes of the three positive controls. Considerable proportions of their genome sequences also constituted RIP mutations. For both the positive and negative controls, analysis at lower stringencies allowed for the identification of many more LRARs and windows suggestive of RIP. Also, as with the simulated data, RIP product parameters did not affect the RIP statistics much, but fewer windows suggestive of RIP were detected in the genomes of the negative controls. Therefore, based on these findings, windows indicating RIP mutations with product index values above 1.1 and substrate index values below 0.75 were implemented on all further analyses performed in this study.

### Validation of RIP analyses using The RIPper

**Figure 3:**
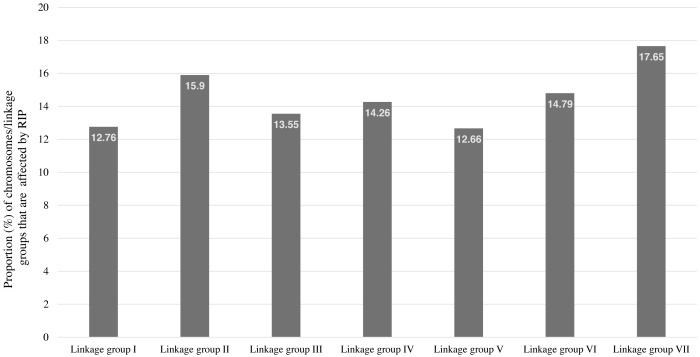
The total percentage of *Neurospora crassa* chromosomes affected by RIP. The total percentage of each chromosome/linkage group of *Neurospora crassa* strain OR74a affected by RIP. These estimates represent the proportion of RIP-positive windows identified in each chromosome sequence.

RIP activity has been experimentally and computationally verified in the *N. crassa* genome (Clutterbuck, 2011; Cambareri et al., 1989). Therefore, The RIPper was applied to the genome assembly of *N. crassa* for calculating genome-wide RIP statistics, to identify putative LRARs, and to determine the extent of RIP mutations in each linkage group (chromosome) of this fungus. Genome-wide RIP analyses showed that a considerable proportion of the *N. crassa* genome assembly (15.26%) constitutes RIP mutations (Table 1). A large number of RIP affected windows (12,545), and numerous LRARs (435) (Table 2 and Table S3) were recorded throughout this genome. Extensive RIP affected windows further showed a reduced GC content compared to that of the remainder of the genome (Table 1). The total percentage of each linkage group of *N. crassa* affected by RIP varied from 12.66% (linkage group I) to 17.65% (linkage group VII) (Fig. 3).

**Table 1:** Genome-wide RIP statistics for *Neurospora crassa* strain OR74a, which was generated using The RIPper.

Genome size (bp)	41,102,378
Total number of windows investigated	82,204
GC content of entire genome assembly (%)	43.34
Total number of RIP positive windows	12,545
Total estimated genome-wide RIP (%)^a^	15.26
Number of LRARs^b^	439
Average size of LRARs (bp)	13,279.08
Average GC content of LRARs (%)	16.04
Sum of all LRARs (bp)	5,829 515
RIP product index^c^ average for LRARs	1.59
RIP substrate index^d^ average for LRARs	0.44
RIP composite index^e^ average for LRARs	1.14

**Notes.**

a Proportion of the genome affected by RIP. Calculated using the total number of windows with RIP-positive index values against the total number of windows investigated for the entire genome sequence.

b LRAR = Putative Large RIP affected genomic regions. More than 4,000 bp that are consecutively affected by RIP.

c Product Index Value [TpA/ApT]: *x* > 1.1.

d Substrate Index Value [CpA + TpG/ ApC + GpT]: 0.75 ≥ *x*.

e Composite Index Value [(TpA/ApT)–(CpA + TpG/ApC + GpT)]: *x* > 0.

**Table 2:** Statistics determined with The RIPper for the first 10 Large RIP Affected Regions (LRARs) identified on chromosome 1 of *Neurospora crassa* strain OR74a.^a^

**LRAR**	**Start (bp)**	**End (bp)**	**Size (bp)**	**Number of consecutive RIP-affected windows**	**RIP indices**			**% GC**
					**Product**^b^	**Substrate**^c^	**Composite**^d^	
1	21,500	34,000	12,500	24	1.67	0.4	1.26	18.37
2	63,000	77,000	14,000	27	1.5	0.47	1.04	14.83
3	77,000	87,000	10,000	19	1.56	0.4	1.16	15.07
4	199,500	208,500	9,000	17	1.78	0.41	1.37	15.5
5	267,000	277,500	10,500	20	1.52	0.55	0.97	18.16
6	278,500	284,000	5,500	10	1.36	0.58	0.77	15.67
7	284,000	288,500	4,500	8	1.52	0.58	0.94	17.56
8	670,500	696,500	26,000	51	1.52	0.44	1.08	14.42
9	872,500	877,500	5,000	9	1.35	0.5	0.85	11.96
10	878,000	905,500	27,500	54	1.46	0.44	1.03	15.05

**Notes.**

a See Table S3 for the full list of putative LRARs on the whole genome of this fungus.

b Product Index Value [TpA/ApT]: *x* > 1.1.

c Substrate Index Value [CpA + TpG/ApC + GpT]: 0.75 ≥ *x*.

d Composite Index Value [(TpA/ApT)–(CpA + TpG/ApC + GpT)]: *x* > 0.

**Figure 4:**
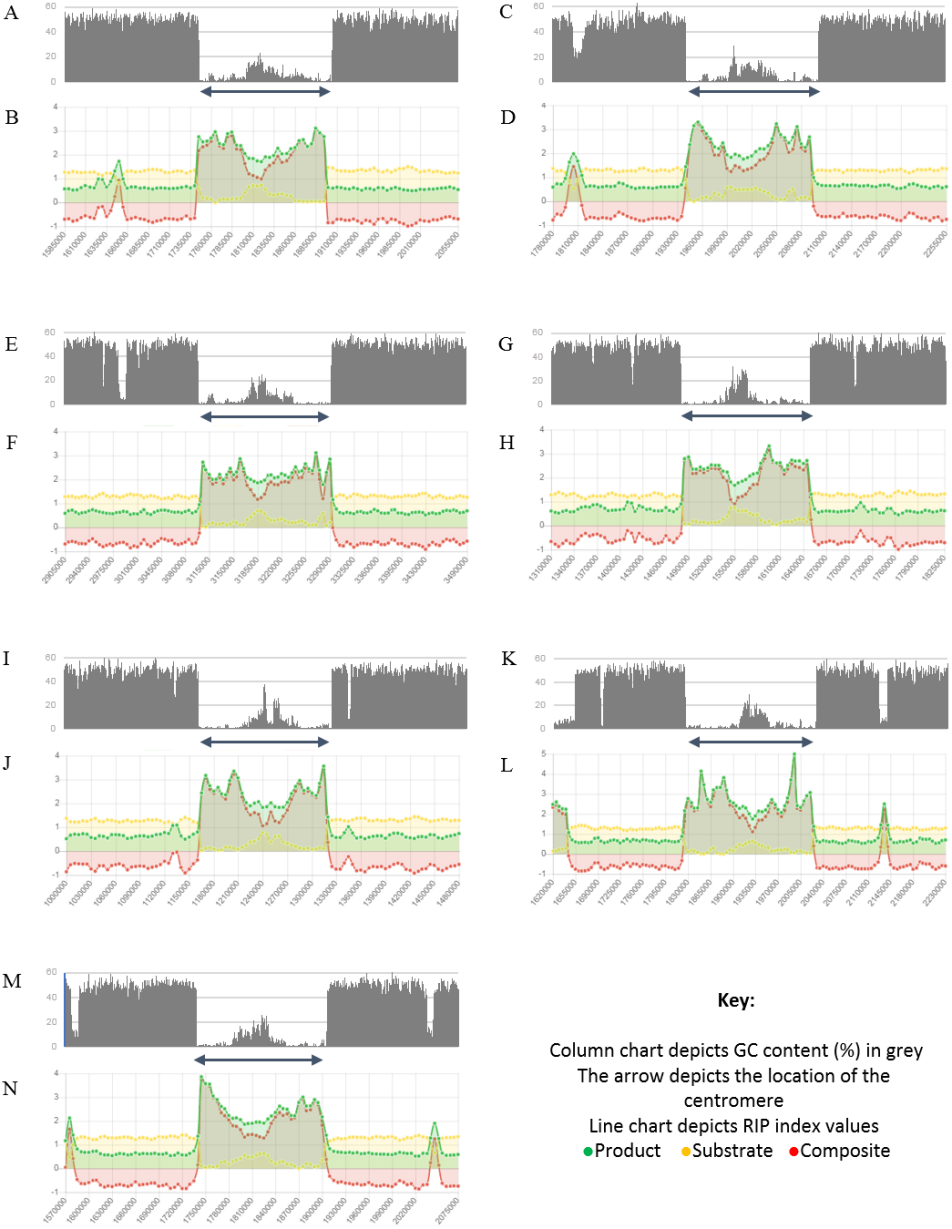
Summary of changes in RIP index values and GC content, of the centromeric regions of the chromosomes of * Trichodermareesei*. The figure depicts changes of the RIP index values and GC content across the centromeric regions of chromosomes of * T. reesei* strain QM6a. The position of the centromeric regions are indicated with an arrow. The bar charts illustrate changes in GC content (%) and the line charts illustrate changes in RIP index values for chromosome 1 (A and B), chromosome 2 (C and D), chromosome 3 (E and F), chromosome 4 (G and H), chromosome 5 (I and J), chromosome 6 (K and L), and chromosome 7 (M and N), respectively. RIP analyses were performed using a 1,000 bp window size and 500 bp step size.

Analysis of the *T. reesei* genome with The RIPper showed a distinctive pattern of GC content and RIP mutations across each of the seven chromosomes of this fungus (Fig. 4, Table 3). The genomic regions corresponding to the centromeres of the *T. reesei* chromosomes were previously suggested to be prone to RIP mutations (Li et al., 2017). In agreement with these previous findings, reduced GC content, together with RIP index values indicating RIP activity, were observed across the length of the seven centromeric genomic regions of this fungus.

**Table 3:** Statistics determined with The RIPper for the centromeric regions of *Trichoderma reesei* (QM6a).

**Replicon**	**Location**	**Length (bp)**	**RIP indices**			**% GC**
			**Product**^a^	**Substrate**^b^	**Composite**^c^	
Chromosome 1	3,104,000–3 303,000	199,000	2.44	0.20	2.24	4.57
Chromosome 2	1,940,500–2,103,000	162,500	2.32	0.11	2.21	4.76
Chromosome 3	1,744,500–1,906,000	161,500	2.25	0.12	2.13	4.49
Chromosome 4	1,482,500–1,659,000	176,500	2.25	0.09	2.1	4.79
Chromosome 5	1,164,500–1,329,000	164,500	2.25	0.27	1.98	4.72
Chromosome 6	1,825,500–2,034,000	208,500	2.48	0.09	2.39	4.16
Chromosome 7	1,741,000–1,914,000	173,000	2.33	0.13	2.19	4.80

**Notes.**

a Product Index Value [TpA/ApT]: *x* ≥ 1.1.

b Substrate Index Value [CpA + TpG/ApC + GpT]: 0.75 ≥*x*.

c Composite Index Value [(TpA/ApT)–(CpA + TpG/ApC + GpT)]: *x* > 0.

**Figure 5:**
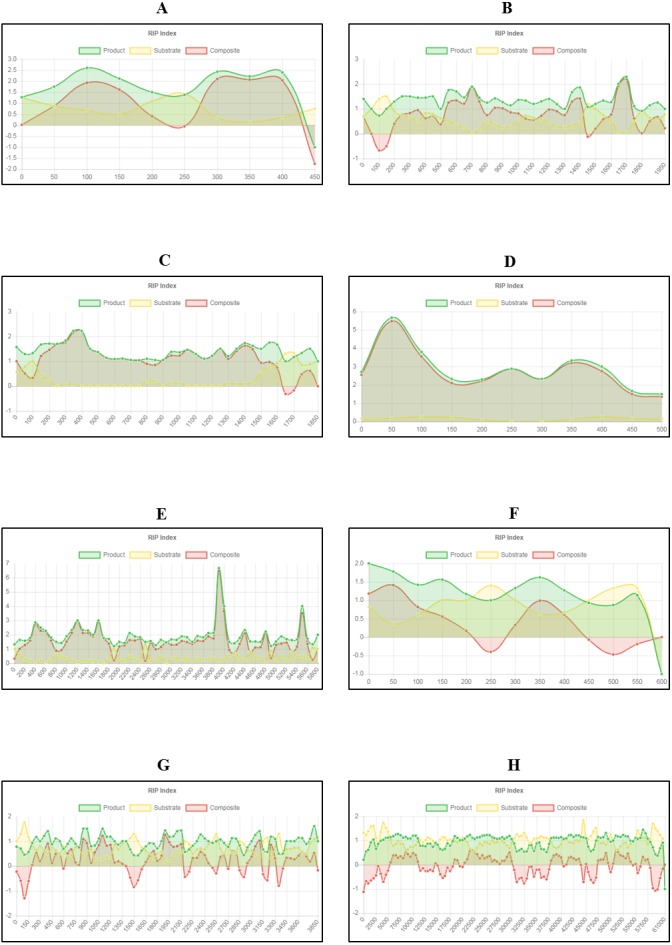
Fine-scale RIP analyses of known fungal sequences affected by RIP. Changes in RIP index values were calculated and graphs generated using The RIPper for fungal sequences known to be affected by RIP (see Table 4). RIP index values illustrated for sequences: (A) *Leptosphaeria maculans* AvrLm4-7 pseudogene; (B) *Neurospora crassa* Punt transposon and 5S ribosomal RNA pseudogene; (C) *Podospora anserina* Fot1-like DNA transposon Pat; (D) *Colletotrichum cereal* retrotransposon Collect1; (E) *Aspergillus fumigatus* long terminal repeat, pol gene; (F) *A. oryzae* transposase (Tan1) gene; (G) *Magnaporthe grisea* retrotransposon, reverse transcriptase and integrase region; and (H) *Chrysoporthe deuterocubensis* retrotransposon and mating type locus. Changes in RIP index values are represented on the y-axis and nucleotide position on the sequence is indicated on the x-axis (indicating the start position of the window). RIP analyses were performed using a 100 bp window size and 50 bp step size for figures A to G, and 1,000 bp window size and 500 bp step size for figure H.

Query sequences for RIP analyses were retrieved based on previously published data (Margolin et al., 1998; Plissonneau et al., 2016; Hamann, Feller & Osiewacz, 2000; Crouch et al., 2008; Paris & Latgé, 2001; Montiel, Lee & Archer, 2006; Nakayashiki et al., 1999; Kanzi et al., 2019). Our results for these gene regions and TE sequences showed an increase in RIP product dinucleotide frequencies (RIP product index), coupled with reduced frequency in dinucleotides targeted by RIP (RIP substrate index) (Fig. 5). RIP was recorded in the AvrLm4-7 pseudogene of *L. maculans*, the 5S rRNA pseudogene of *N. crassa*, the DNA transposon of *P. anserina*, retrotransposon sequences of *C. cereale*, *M. grisea, C. deuterocubensis,* and within long terminal repeat sequences of *A. fumigatus.* Moreover, regional variation in the extent of RIP was observed in the *M. grisea* and *C. deuterocubensis* sequences. Some windows were RIP affected, while others remained unchanged by RIP. In comparison, the particular nucleic acid sequences of *L. maculans*, *N. crassa*, *P. anserina*, *C. cereale*, *A. fumigatus*, and *A. oryzae* were more extensively RIP affected across the length of the sequences investigated. The occurrence overall of less RIP in the *M. grisea* and *C. deuterocubensis* sequences were further reflected in the summary of RIP statistics (Table 4). Overall, extensively RIP affected sequences had RIP index values indicating strong RIP responses that are coupled with reduced GC content (Table 4). Conversely, the sequences with a reduced RIP response had higher GC content averages. These results were all in agreement with previous findings (Margolin et al., 1998; Plissonneau et al., 2016; Hamann, Feller & Osiewacz, 2000; Crouch et al., 2008; Paris & Latgé, 2001; Montiel, Lee & Archer, 2006; Nakayashiki et al., 1999; Kanzi et al., 2019), which suggests that the fine-scale RIP analyses implemented in The RIPper is valuable for identifying specific RIP affected regions.

**Table 4:** Statistics determined with The RIPper for genomic regions that were previously reported to be affected by RIP.

**Species**	**Description of the genomic region**	**Length (bp)**	**RIP indices**			**% GC**	**References**
			**Product**^a^	**Substrate**^b^	**Composite**^c^		
*Leptosphaeria maculans*	AvrLm4-7 pseudogene	478	1.67	0.74	0.93	35.23	Plissonneau et al. (2016)
*Neurospora crassa*	Transposon Punt (complete sequence) and 5S ribosomal RNA pseudogene (partial sequence)	1,994	1.35	0.59	0.76	35.12	Margolin et al. (1998)
*Podospora anserina*	Degenerate Fot1-like DNA transposon Pat	1,860	1.4	0.31	1.09	28.47	Hamann, Feller & Osiewacz (2000)
*Colletotrichum cereale*	Isolate PA-50005 clone I29 retrotransposon Collect1 (partial sequence)	549	2.87	0.15	2.72	28.52	Crouch et al. (2008)
*Aspergillus fumigatus*	Long terminal repeat (complete sequence) and pol gene (partial sequence)	5,820	1.94	0.38	1.56	30.91	Paris & Latgé (2001)
*Aspergillus oryzae*	Clone 2 putative transposase (Tan1) gene (partial sequence)	601	1.16	0.78	0.38	32.87	Montiel, Lee & Archer (2006)
*Magnaporthe grisea*	Retrotransposon DNA, reverse transcriptase and integrase region	3,895	0.97	0.72	0.25	41.99	Nakayashiki et al. (1999)
*Chrysoporthe deuterocubensis*	Retrotransposon and mating type locus	61,000	1.1	1.13	0.12	40.94	Kanzi et al. (2019)

**Notes.**

a Product Index Value [TpA/ApT]: *x* > 1.1.

b Substrate Index Value [CpA + TpG/ApC + GpT]: 0.75 ≥ *x*.

c Composite Index Value [(TpA/ApT)–(CpA + TpG/ApC + GpT)]: *x* > 0.

## Discussion

Here we present The RIPper as a web-based set of tools for genome-wide investigation of RIP in fungi in the phylum Ascomycota. This tool is user friendly, open access and allows for detailed investigation of RIP mutations on a genome-wide scale. Automation of RIP index calculations provides information on the extent of putative RIP mutations acquired for a given fungal genome sequence, in a time-efficient and reproducible manner. A RIP profile provides a summary of RIP statistics, while fine-scale analysis of RIP index values enables detailed investigations of RIP activity in genomic regions of interest. These profiles also provide an innovative avenue toward assessing RIP capability among fungi. The user defined criteria and availability of all the RIP data generated by The RIPper afford the user flexibility to fine-tune the stringency at which analyses are performed. The RIPper significantly complements current *in silico* approaches to measure RIP and to identify genomic regions affected by RIP.

RIP analyses performed with The RIPper allowed validation of this tool’s ability to identify RIP affected regions in fungal sequences. Comparable to previous findings (Testa, Oliver & Hane, 2016), a large proportion of the genome assembly and of each individual linkage group of *N. crassa* were indicated to be affected by RIP. Similarly, the centromeres of *T. reesei* displayed reduced GC content and, as previously suggested (Li et al., 2017), these regions were characterized by a strong RIP signal (Li et al., 2017). Moreover, our results showed that RIP likely drives the formation of long stretches of genomic regions affected by RIP with reduced GC content. Fine-scale RIP analyses conducted with The RIPper further allowed identification of the consequences of RIP in gene regions and gene sequences known to be targeted by this pathway (Margolin et al., 1998; Plissonneau et al., 2016; Hamann, Feller & Osiewacz, 2000; Crouch et al., 2008; Paris & Latgé, 2001; Montiel, Lee & Archer, 2006; Nakayashiki et al., 1999; Kanzi et al., 2019). Overall, extensively RIP affected sequences had RIP index values indicative of strong RIP signal that were coupled with reduced GC content.

The findings of this study suggest that use of more stringent RIP parameters can reduce the occurrence of false positive results (i.e., regions erroneously suggested to contain RIP mutations). The use of simulated sequences allowed optimization of RIP analysis parameters, the use of which reduced the influence of GC content composition on RIP statistics. Furthermore, the use of these more stringent parameters allowed the detection of RIP mutations in organisms known to be RIP capable, while these mutations were absent from the sequences of species incapable of acquiring transition mutations due to RIP. Therefore, although the parameters applied for RIP analyses using The RIPper may be adjusted according to the specifications of the user, our results show RIP substrate index values of ≤ 0.75 and RIP product index values of ≥ 1.1 are most likely to provide an accurate reflection of the occurrence and extent of RIP mutations in fungal sequences.

Data generated using The RIPper provides valuable insights on the potential occurrence of RIP as well as how this pathway may influence gene and genome evolution. For example, RIP is known to be an important driver of effector and avirulence gene evolution in fungi (Rouxel et al., 2011; Fudal et al., 2009; Meerupati et al., 2013). RIP driven mutation of the effector gene of *L. maculans*, AvrLm4-7, for example, was credited as contributing to overcoming host plant resistance (Plissonneau et al., 2016). Additionally, retrotransposon integration followed by RIP activity probably contributed to the evolution of the mating type locus of *C. deuterocubensis* (Kanzi et al., 2019), where RIP mutations likely affect reproductive strategies of this fungus (Kanzi et al., 2019). Therefore, RIP not only serves to counteract the deleterious consequences of TEs, but also contributes to the evolution of gene regions underlying important aspects of biology and host range (Rouxel et al., 2011; Meerupati et al., 2013; Kanzi et al., 2019).

Genome-wide RIP analyses provides information on the occurrence and the potential extent of RIP mutations in fungal sequences. Moreover, *in silico* RIP assessment using The RIPper can be done without any prior knowledge of TE content of a genome. Additionally, accurate alignment of degraded repeated regions is not always possible, thus limiting the effectiveness of alignment-based RIP analyses. The RIPper allows for adjustable parameters in order to identify potentially RIP affected regions, while other *in silico* approaches employing a sliding window approach (Hane & Oliver, 2008) are limited to the parameters associated with a single species (*N. crassa*) (Hane & Oliver, 2008). The application of such parameters to other fungi may lead to false positive identification of RIP affected regions (see Table S2).

Despite the advantages of using The RIPper, the *in silico* assessment of RIP in fungal sequences using this tool and other tools is associated with a number of limitations. These include the uncertainty of the exact boundary of a RIP affected genomic region when a sliding-window approach is implemented (Testa, Oliver & Hane, 2016). Moreover, low-quality genome assemblies with low coverage often exclude much of the repetitive AT-rich genomic regions in the genome. These regions in RIP capable ascomycetes typically reflect a large proportion RIP affected content of these genomes. Therefore, the use of low-quality genome assemblies my lead to an underestimation of the true occurrence and extent of RIP mutations.

Data generated using The RIPper should serve to complement existing *in silico* techniques for measuring RIP mutations. For example, OcculterCut (Testa, Oliver & Hane, 2016) can provide evidence on genome segmentation based on altered GC content due to RIP. RIPCAL alignment-based analyses (Hane & Oliver, 2008) provides information on the dominant RIP mutation form in classes of repeated sequences. RIPCAL and OcculterCut analyses provide information on the nucleotide composition of RIP affected regions. Therefore, The RIPper serves to provide a further line of evidence to study the occurrence and the extent of RIP mutations in fungi. Application of The RIPper and other *in silico* techniques will undoubtedly aid our current understanding of not only RIP capability, but also the impact of this pathway on the evolutionary dynamics of Ascomycota.

## Conclusion

Here we present The RIPper as a web-based set of tools for genome-wide investigation of RIP in fungi in the phylum Ascomycota. This tool is user friendly, open access and allows for detailed investigation of RIP mutations on a genome-wide scale. Automation of RIP index calculations provides information on the extent of putative RIP mutations acquired for a given fungal genome sequence, in a time-efficient and reproducible manner. A RIP profile provides a summary of RIP statistics, while fine-scale analysis of RIP index values enables detailed investigations of RIP activity in genomic regions of interest. These profiles also provide an innovative avenue toward assessing RIP capability among fungi. The user defined criteria and availability of all the RIP data generated by The RIPper afford the user flexibility to fine-tune the stringency at which analyses are performed. The RIPper significantly complements current *in silico* approaches to measure RIP and to identify genomic regions affected by RIP.

##  Supplemental Information

10.7717/peerj.7447/supp-1 Figure S1 Total percentage of nucleic acid sequence affected by RIP Bar charts summarizing the total proportion of simulated nucleic acid sequences (1 Mbp) that constitutes RIP mutations. Calculated using different RIP parameters ^1,2^ for a given GC content range ^3^. ^1^ RIP product index value cut-off: 1.1, 1.15 1.2, and 1.25 ^2^ RIP substrate index cut-off: 0.75, 0.8, 0.85, and 0.9 ^3^ The average GC content (10%, 20%, 30%, 40%, 50%, 60%, 70%, 80%, and 90%) of 1Mbp simulated nucleic acid sequences consists 100 replicates of randomly generated data.

10.7717/peerj.7447/supp-2 Table S1 Genome-wide RIP index values of *Neurospora crassa* Example of results using the RIP Genome tool of The RIPper for the genome-wide RIP analyses of the *Neurospora crassa* (Strain:OR74a; assembly number: GCA_000182925.2; accessed through the National Centre for Biotechnology Information (NCBI; https://www.ncbi.nlm.nih.gov)) genome assembly. The results also includes the average GC content calculated per window. RIP product index values above 1.1, RIP substrate index values below 0.9, and RIP composite index values above 0 indicate RIP affected windows (Selker, 1990; Selker et al., 2003; Lewis et al., 2009; Margolin et al., 1998).

10.7717/peerj.7447/supp-3 Table S2 RIP statistics (RIP product and substrate cut-off values) calculated for nine datasets that were simulated based on GC content datasets Highlighted cells illustrate the instances where the average RIP affected proportion (%) of dataset is greater than 1%.

10.7717/peerj.7447/supp-4 Table S3 Genome-wide RIP statistics of control organisms investigated using different RIP index cut-off values Values highlighted in blue indicate a larger proportion of the overall recorded genome-wide RIP of negative control organisms, due to less stringent RIP substrate index value parameters.

10.7717/peerj.7447/supp-5 Supplemental Information 1 Alternate Fig. 2 with colors adjusted for accessibility

10.7717/peerj.7447/supp-6 Supplemental Information 2 Alternate Fig. 4 with colors adjusted for accessibility

10.7717/peerj.7447/supp-7 Supplemental Information 3 Alternate Fig. 5 with colors adjusted for accessibility

## Additional Information and Declarations

### Data Availability

The following information was supplied regarding data availability: The code is available from GitHub:
https://github.com/TheRIPper-Fungi/TheRIPper.
