# Supplementary material for: The RIPper, a web-based tool for genome-wide quantification of Repeat-Induced Point (RIP) mutations
Source: PeerJ. 2019 Aug 26;7:e7447. doi: 10.7717/peerj.7447 (PMC6714961; doi:10.7717/peerj.7447)
Supplement: Figure S1 — Bar charts summarizing the total proportion of simulated nucleic acid sequences (1 Mbp) that constitutes RIP mutations. Calculated using different RIP parameters 1,2 for a given GC content range 3. 1 RIP product index value cut-off: 1.1, 1.15 1.2, and 1.25 2 RIP substrate index cut-off: 0.75, 0.8, 0.85, and 0.9 3 The average GC content (10%, 20%, 30%, 40%, 50%, 60%, 70%, 80%, and 90%) of 1Mbp simulated nucleic acid sequences consists 100 replicates of randomly generated data. [file peerj-07-7447-s001.pdf]

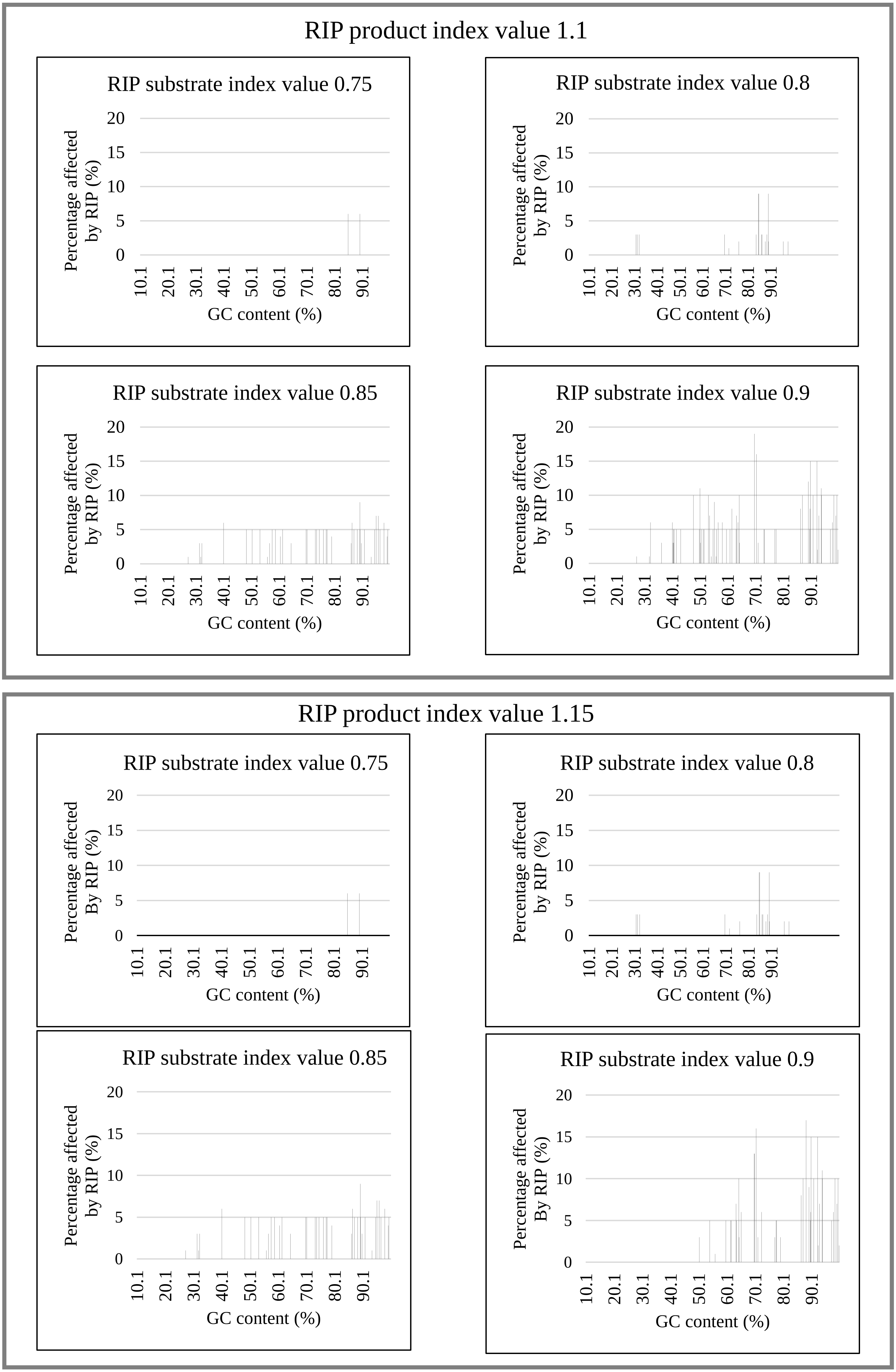

RIP product index value 1.1
RIP substrate index value 0.75
RIP substrate index value 0.8
RIP substrate index value 0.85
RIP substrate index value 0.9
Percentage affected by RIP (%)
GC content (%)
0
5
10
15
20
10.1
20.1
30.1
40.1
50.1
60.1
70.1
80.1
90.1
RIP product index value 1.15
RIP substrate index value 0.75
RIP substrate index value 0.8
RIP substrate index value 0.85
RIP substrate index value 0.9
Percentage affected By RIP (%)
Percentage affected by RIP (%)
GC content (%)

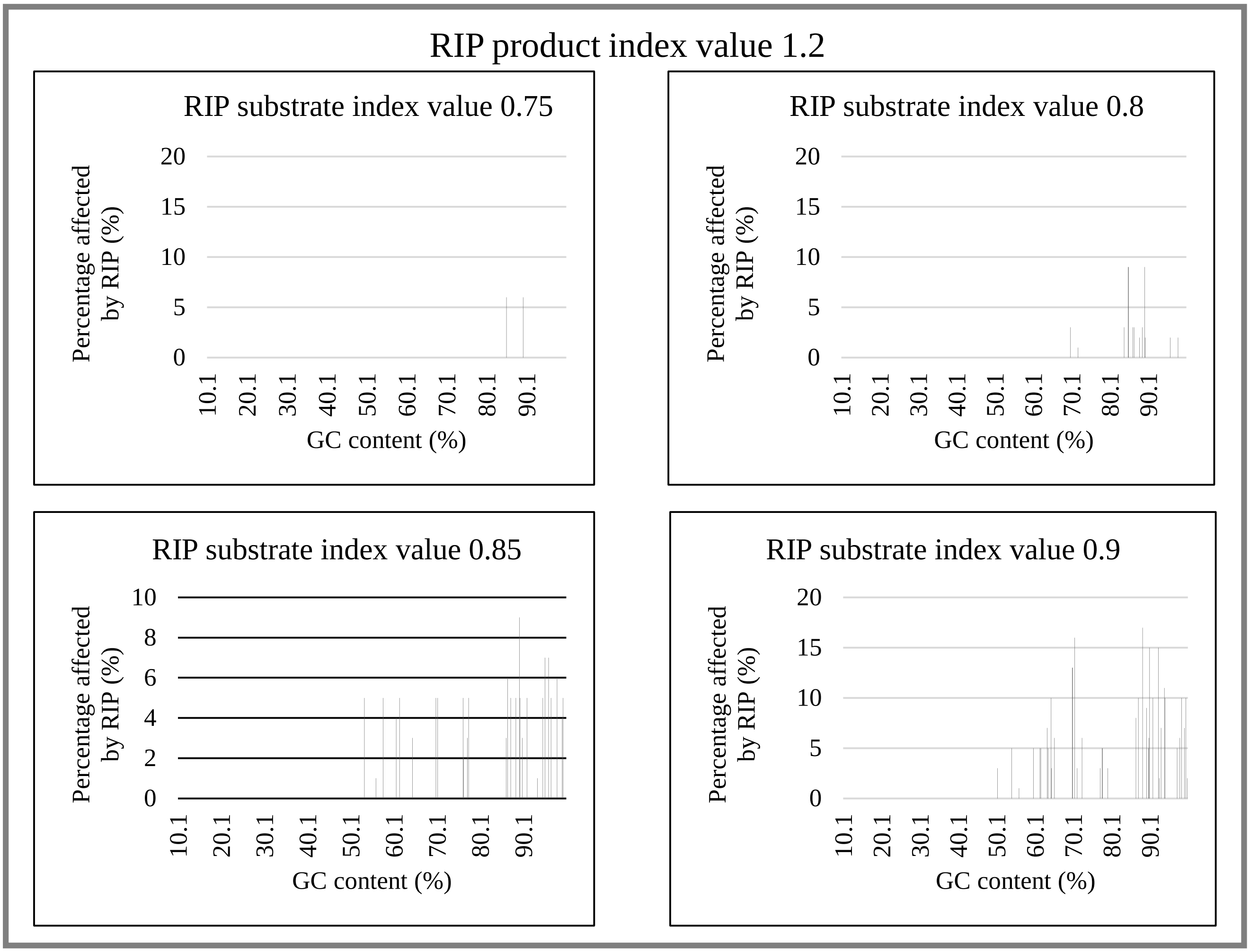
RIP product index value 1.2
RIP substrate index value 0.75
RIP substrate index value 0.8
RIP substrate index value 0.85
RIP substrate index value 0.9
Percentage affected by RIP (%)
GC content (%)
10.1 20.1 30.1 40.1 50.1 60.1 70.1 80.1 90.1

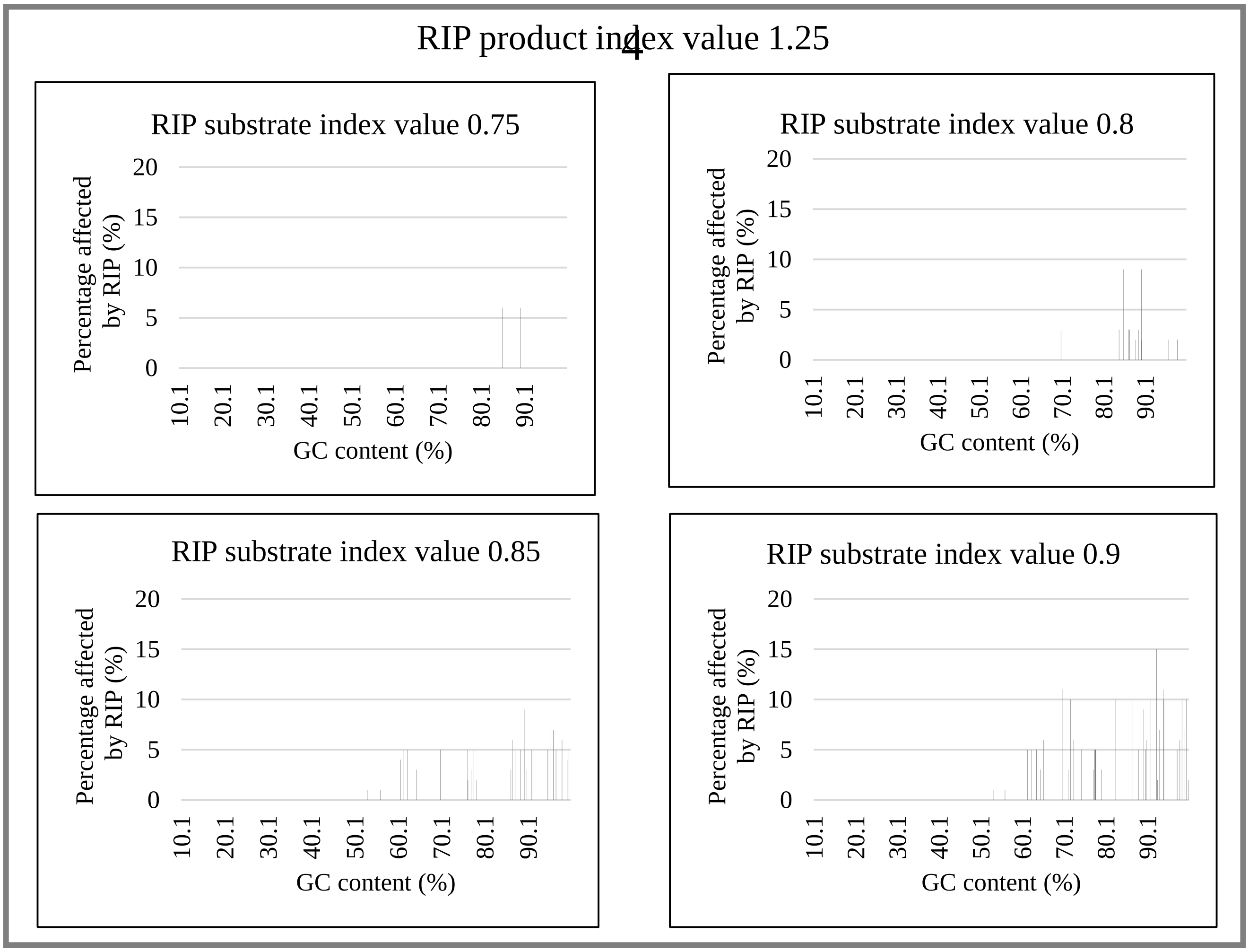
RIP product index value 1.25
RIP substrate index value 0.75
RIP substrate index value 0.8
RIP substrate index value 0.85
RIP substrate index value 0.9
Percentage affected by RIP (%)
GC content (%)
10.1 20.1 30.1 40.1 50.1 60.1 70.1 80.1 90.1
