# Supplementary figures and images for: The RIPper, a web-based tool for genome-wide quantification of Repeat-Induced Point (RIP) mutations
Source: PeerJ. 2019 Aug 26;7:e7447. doi: 10.7717/peerj.7447 (PMC6714961; doi:10.7717/peerj.7447)

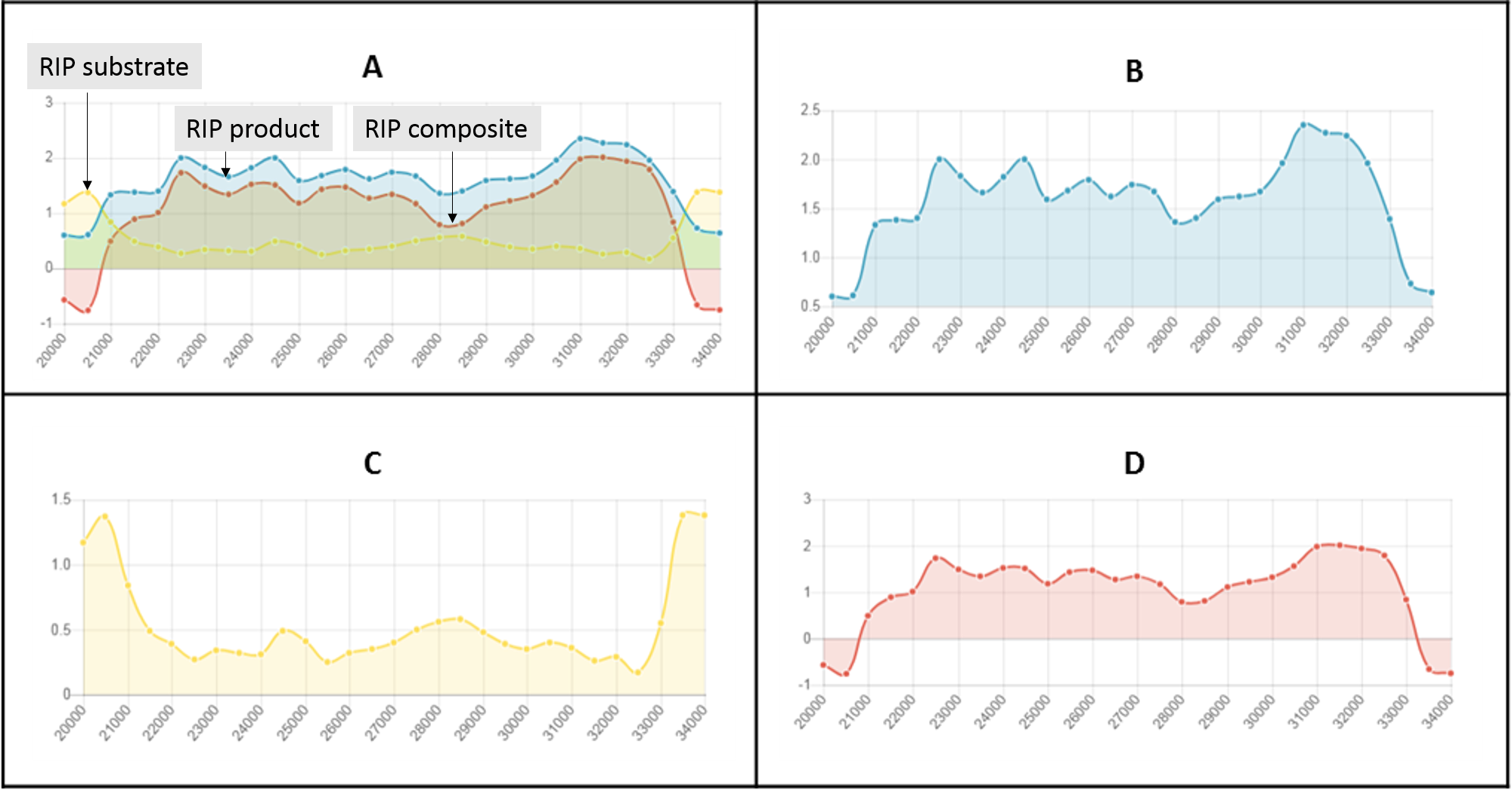

Supplement: Supplemental Information 1 [file peerj-07-7447-s005.png]

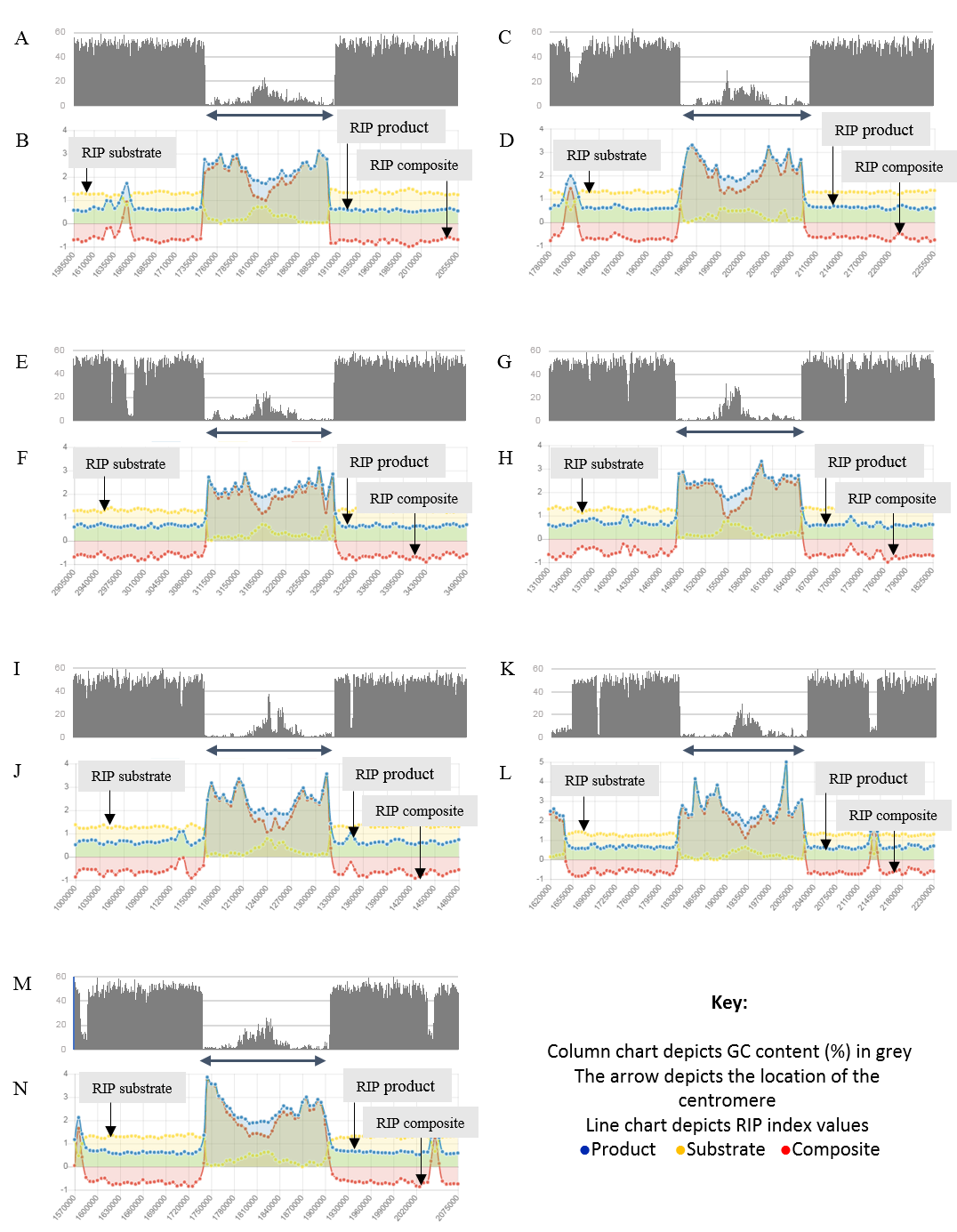

Supplement: Supplemental Information 2 [file peerj-07-7447-s006.png]

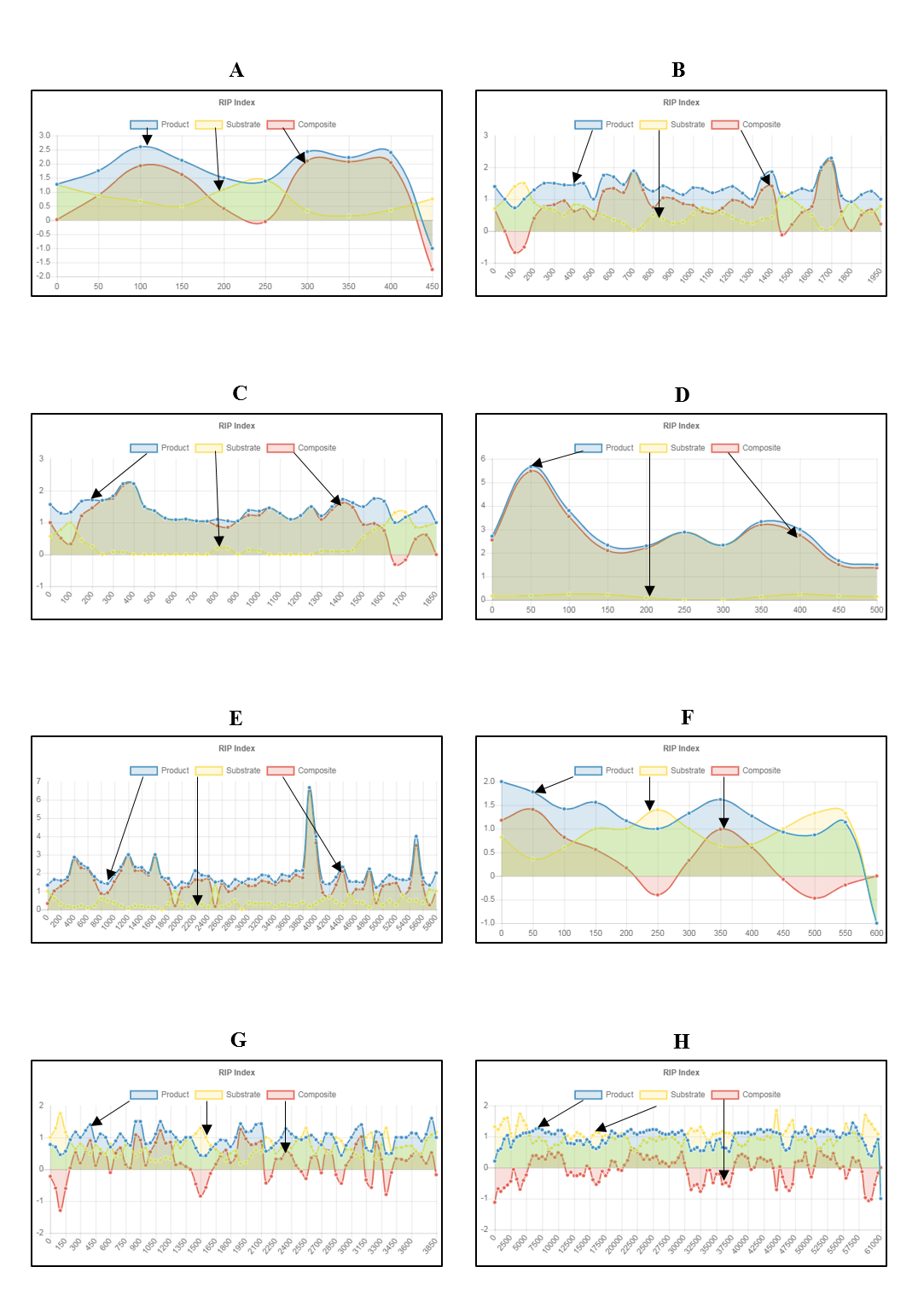

Supplement: Supplemental Information 3 [file peerj-07-7447-s007.png]
